# Novel cerebrospinal fluid anti-central nervous system IgG antibodies can identify immunotherapy-responsive neuropsychiatric disorders

**DOI:** 10.3389/fimmu.2025.1612844

**Published:** 2025-07-07

**Authors:** Hannah Preßler, Isabel Bünger, Harald Prüss

**Affiliations:** ^1^ Department of Neurology and Experimental Neurology, Charité-Universitätsmedizin Berlin, Berlin, Germany; ^2^ German Center for Neurodegenerative Diseases (DZNE) Berlin, Berlin, Germany; ^3^ Department of Pediatric Neurology, Charité-Universitätsmedizin Berlin, Berlin, Germany

**Keywords:** seronegative autoimmune encephalitis, autoimmune neurology and psychiatry, immunotherapy, novel anti neuronal autoantibodies, immunofluorescence

## Abstract

**Background:**

Autoantibodies (Abs) targeting the central nervous system (CNS) can cause various neuropsychiatric autoimmune diseases. The potential response to immunotherapy necessitates the continuous expansion of Ab testing strategies including non-antigen-specific screening assays. This study investigated whether tissue-based screening using unfixed murine CNS sections can help to identify patients with immunotherapy-responsive neuropsychiatric diseases after routine Ab panels yielded negative results.

**Methods:**

This retrospective single-center study screened cerebrospinal fluid (CSF) of 279 patients for immunoglobulin G (IgG) anti-CNS Abs using unfixed mouse brain. Patients had a variety of neuropsychiatric conditions, in which an autoimmune contribution was considered. Previous testing for a panel of established autoantibodies using cell-based assays remained negative. Of 238 patients, paired serum samples were available.

**Results:**

A subgroup of 55 patients (20%) showed novel anti-CNS autoantibody patterns in CSF, consisting of anti-myelin (n=13), anti-neuropil (n=14), anti-vessel (n=8), anti-tight junction (n=5), anti-cellular (n=8), and anti-astroglial (n=7) autoantibodies. Thirty-six patients (65%) fulfilled criteria for possible, probable, or definite autoimmune encephalitis or paraneoplastic neurological syndrome. Memory impairment (73%) and psychiatric abnormalities (64%) were the most frequent symptoms. Antibody subtypes were not significantly associated with clinical parameters at this sample size, however, there was a trend towards better response to immunotherapy with antibodies against myelin, neuropil, and neuronal cells, while patients with anti-vessel antibodies did not improve. CNS autoantibodies mainly disappeared parallel to clinical improvement. In 46% of treated patients, physicians would not have started immunotherapy without detection of anti-CNS autoantibodies, and the vast majority of patients stabilized or improved.

**Conclusion:**

Novel CNS Abs were common in patients with suspected ‘seronegative’ autoimmune neuropsychiatric disorders. Detection facilitated identification of immunotherapy-responsive cases and enabled treatment initiation without increasing unnecessary treatments. Thus, tissue screening using unfixed mouse brain applied in patients with suspected neuropsychiatric autoimmune diseases parallel to established cell-based assays. Future studies should identify the underlying antigens, demonstrate the pathogenic role in animal models, and implement promising Abs into diagnostic routine panels.

## Introduction

Over the past two decades, the discovery of autoantibodies (Abs) against neuronal or glial antigens has transformed the diagnostic and therapeutic landscape of neuropsychiatric disorders (1). An autoimmune contribution is now confirmed in several conditions previously categorized as idiopathic, psychiatric, or infectious (2), which offered new therapeutic options such as Ab-specific targeted immunotherapies (3). Abs are the main disease driver in several forms of autoimmune encephalitis (AE) including NMDA receptor (NMDAR) encephalitis, where they directly cause brain dysfunction (4, 5). In the meantime, NMDAR Abs were also recognized in a broader spectrum of disorders, including psychiatric diseases (6), dementia (7), and cognitive impairment post-stroke (8) or in cancer patients (9, 10). Likewise, in IgLON5 disease, Abs contribute to a heterogeneous spectrum of symptoms covering autoimmune and neurodegenerative mechanisms, ranging from sleep disturbances to bulbar dysfunction to impaired cognition (11, 12). Thus, Abs have the potential to contribute to pathology across a spectrum of seemingly unrelated diseases, highlighting their role as disease modulators, comparable to genetics, lifestyle, metabolic or environmental risk factors (3).

Given the importance of positive Ab findings for clinical decision-making and the ongoing discovery of further Abs, routine testing may overlook treatable etiologies including AE (13). Most commonly, cell-based assays (CBA) are used to screen for a predefined panel of Abs but can certainly not detect novel Abs. This can delay or prevent immunotherapy in treatment-responsive patients. Recent studies have demonstrated the added diagnostic value of TBA in both CSF and serum in AE workups. TBA can be used as a first screening tool, with follow-up confirmation by CBA depending on the immunofluorescence staining pattern (14) or as a “second-line” approach in specialized laboratories when standard assays are inconclusive (15).

In our tertiary care center at Charité Universitätsmedizin Berlin, we implemented an Ab testing strategy with CBA-based detection using 21 antigens, complemented with unfixed murine CNS tissue-based screening for novel CSF and serum IgG autoreactivity. While tissue-based screening for novel Abs has been applied in prior exploratory studies (16, 17), its use in clinical routine and direct impact on diagnosis and treatment has not been systematically evaluated to date. Therefore, we analyzed the frequency of novel findings, the association with clinical symptoms and how reporting of novel Abs modified treatment decisions in clinical routine.

## Materials and methods

### Patients and data acquisition

Between January 2019 and June 2023, CSF, and serum samples of 279 patients treated at Charité Universitätsmedizin Berlin were screened in our in-house research laboratory for the presence of CNS autoantibodies. Patients of this single-center study had various neurological or psychiatric symptoms for which the treating physicians considered an autoimmune contribution. Patients were seen in the departments of Neurology, Psychiatry, Rheumatology, Pediatrics, neurological intensive care unit (ICU), and outpatient clinics for encephalitis, dementia, epilepsy, and movement disorders. Ethical approval for publication of clinical data was obtained from the Charité ethics committee (#EA2/066/20).

Medical records were reviewed for demographic information, clinical symptoms, applied diagnostics (laboratory tests, CSF findings, imaging, electroencephalography (EEG)), as well as treatment and outcome profiles. Neurofilament (NfL) concentrations in CSF were measured using the SIMOA Nf-light kit (Quanterix Corp, Boston, MA, USA), according to the manufacturer’s protocol (18). Immunotherapies were classified into first line (methylprednisolone, intravenous immunoglobulins (IVIG), plasma exchange (PE), and immunoadsorption (IA)), second line (rituximab, cyclophosphamide, long-term immunosuppressants [IST]) and third line therapies (daratumumab, bortezomib).

The clinical severity of neurological disease was evaluated using chart-based neurological status assessments during ICU or hospital stay or outpatient visits, with retrospective mRS scoring. A favorable outcome was defined as mRS score of 0 to 2, while poor outcome was indicated by an mRS score of 3-6. For definition of clinical cases, we referred to the relevant guidelines, e.g. for autoimmune encephalitis (19), paraneoplastic neurologic syndrome (PNS) (20), autoimmune psychosis (21), possible psychiatric presentations of autoimmune encephalitis (22), or autoimmune epilepsy (23).

### Autoantibody screening

All samples were screened for the presence of anti-CNS autoantibodies using commercial panel tests including line blots, ELISA, and cell-based assays (CBAs) (Labor Berlin GmbH, Berlin, Germany). The panel included Abs against amphiphysin, CV2 (CRMP5), GAD65, Hu, Ri, Yo, PNMA2 (Ma2/Ta), Zic4, SOX1, Tr (DNER), glutamate receptor (AMPAR1/2, NMDAR), DPPX, GABA_B_R, mGluR5, Glycin-R, LGI1, myelin of peripheral nerves, Caspr2, dopamine-2 receptor, MOG, and aquaporin-4.

Samples were additionally screened on an in-house TBA using 20 µm unfixed sagittal mouse brain sections (10–12 weeks-old C57BL/6 male mice) as described previously (16, 24, 25). In brief, mice were sacrified, brains removed, snap-frozen in 2-methylbutane and stores at – 80°C. Then 20 μm sagittal sections were cut using a cryostat, without further fixation. Sections were rinsed in phosphate-buffered saline (PBS) for 5 min and preincubated in blocking solutions (5% normal goat serum, 2% bovine serum albumin (BSA), 0.05% sodium acid, 0.1% Triton X-100). After preincubation for 45 min (RT), CSF samples (200 μL) were added undiluted, sera (200 μL) diluted 1:200 and incubated overnight at 4°C and IgG binding detected with goat anti-human IgGAF488-antibody (Dianova, #109-545-003). Finally, sections were washed (3×, 5 min) in PBS, mounted and coverslipped with Immunomount Medium. Sections were examined via fluorescence microscopy by two independent raters using a semi-quantitative fluorescence score blinded to the clinical information and Ab status of the patients (26, 27).The scale included “0” (negative), “+” (weak) “++” (moderate) and “+++/++++” (positive/strongly positive). Only samples with specific CSF Ab patterns scoring positive or strongly positive were further analyzed for clinical data.

### Statistical analysis

Descriptive statistics are presented with medians and interquartile ranges (25th and 75th percentiles) or frequencies and percentages, as appropriate. Categorical variables were compared with Chi-square tests, Fisher’s exact tests, and odds ratios (ORs) with 95% confidence intervals (CIs) were calculated. The normality of distributions was assessed using the Shapiro-Wilk test; for p-values <0.05, the Mann-Whitney U test was used for non-normally distributed data, and for p-values ≥0.05, the t-test was used for independent samples. Continuous variables were analyzed using the Mann-Whitney U test or independent samples t-test. Statistical analysis and data visualization were performed using IBM SPSS Statistics 29.0, Biorender and Python version 3.9 employing the packages Matplotlib, Pandas, NumPy, SciPy, and Seaborn.

## Results

### Patient cohorts and autoantibody detection

**Figure 1:**
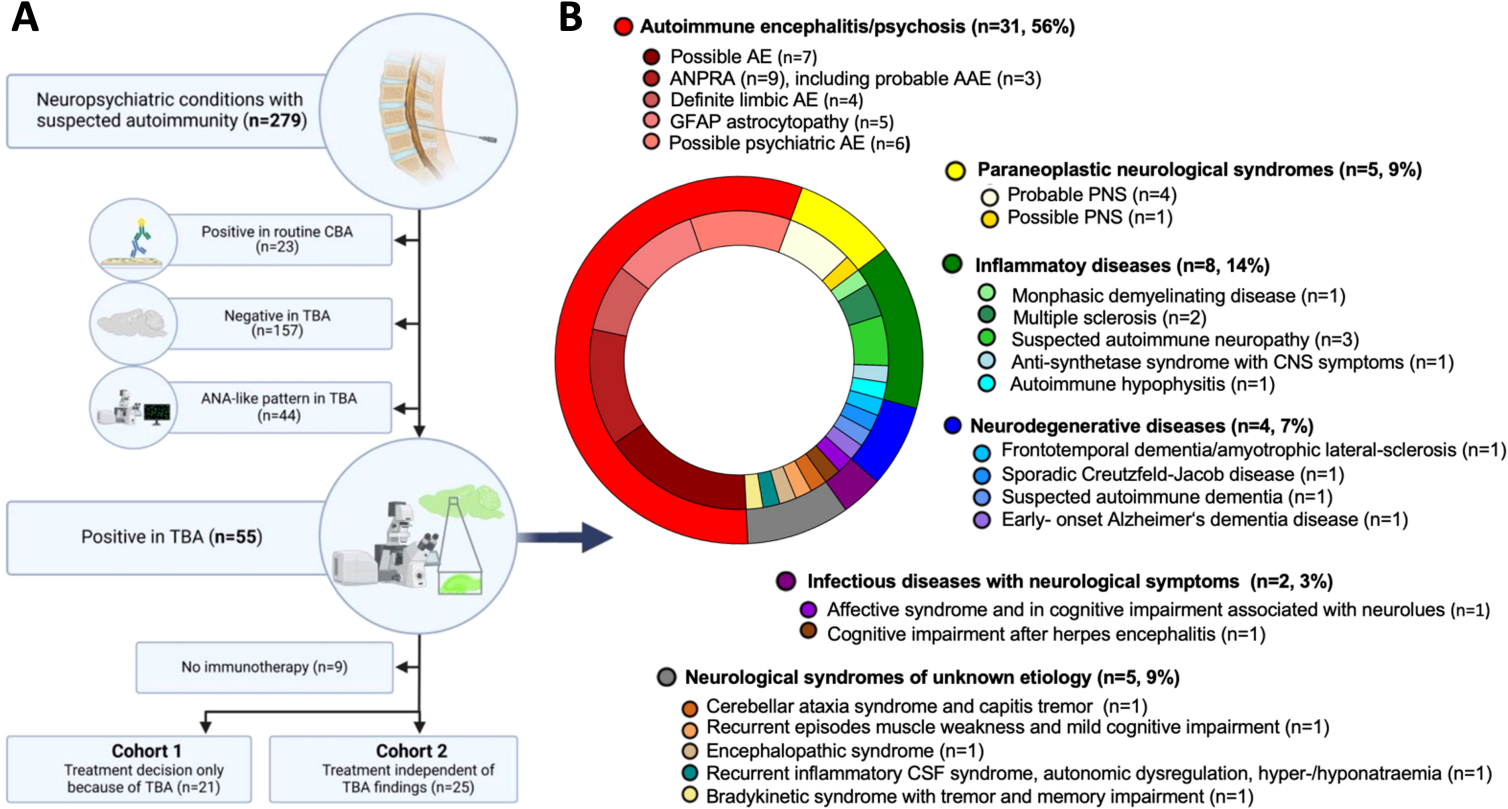
Cohort selection and diagnoses of autoantibody-positive patients **(A)** Patients with various neuropsychiatric disorders and suspected autoimmunity were screened for autoantibodies using commercial panel tests and in-house tissue-based assays (TBAs). Forty-six of 55 patients with novel autoantibody patterns received immunotherapy, of which the decision for immunotherapy was exclusively based on the TBA findings in 21 (*cohort 1*), while treatments would have been initiated in the remaining 25 also without knowledge of these autoantibodies, e.g. due to inflammatory changes in MRI or CSF (*cohort 2*). **(B)** The ring chart illustrates the spectrum of diagnoses among all patients who tested positive for CSF IgG antibodies on unfixed mouse brain sections, excluding patients with anti-nuclear antibodies or positivity already in commercial assays. Ab, antibody; AE, autoimmune encephalitis; ANPRA, autoantibody-negative but probable autoimmune encephalitis; CSF, cerebrospinal fluid; GFAP, glial fibrillary acidic protein; LE, limbic encephalitis; PNS, paraneoplastic neurological syndrome.

We collected CSF samples from 279 patients, of whom 238 also had paired serum samples, in a single-center setting at Charité Universitätsmedizin Berlin. Treating physicians submitted samples as part of the clinical routine as they considered an autoimmune contribution in the differential diagnosis workup (**Figure 1A**). Routine CBA could identify established autoantibodies in 23 patients including antibodies against NMDAR (n=13), LGI1 (n=3), glycine receptor (n=1), GAD65 (n=1), GAD65/Hu/Zic-4 (n=1), mGluR5 (=1), mGluR1 (n=1), IgLON5 (n=1), and DPPX (n=1). Using indirect immunofluorescence on unfixed mouse brain sections, CSF, and serum samples of 157 patients were negative, 44 patients had an anti-nuclear antibody (ANA) pattern (**Figure 1A**), which corresponded to ANA patterns on Hep2 cells and were thus excluded from further analysis.

**Figure 2:**
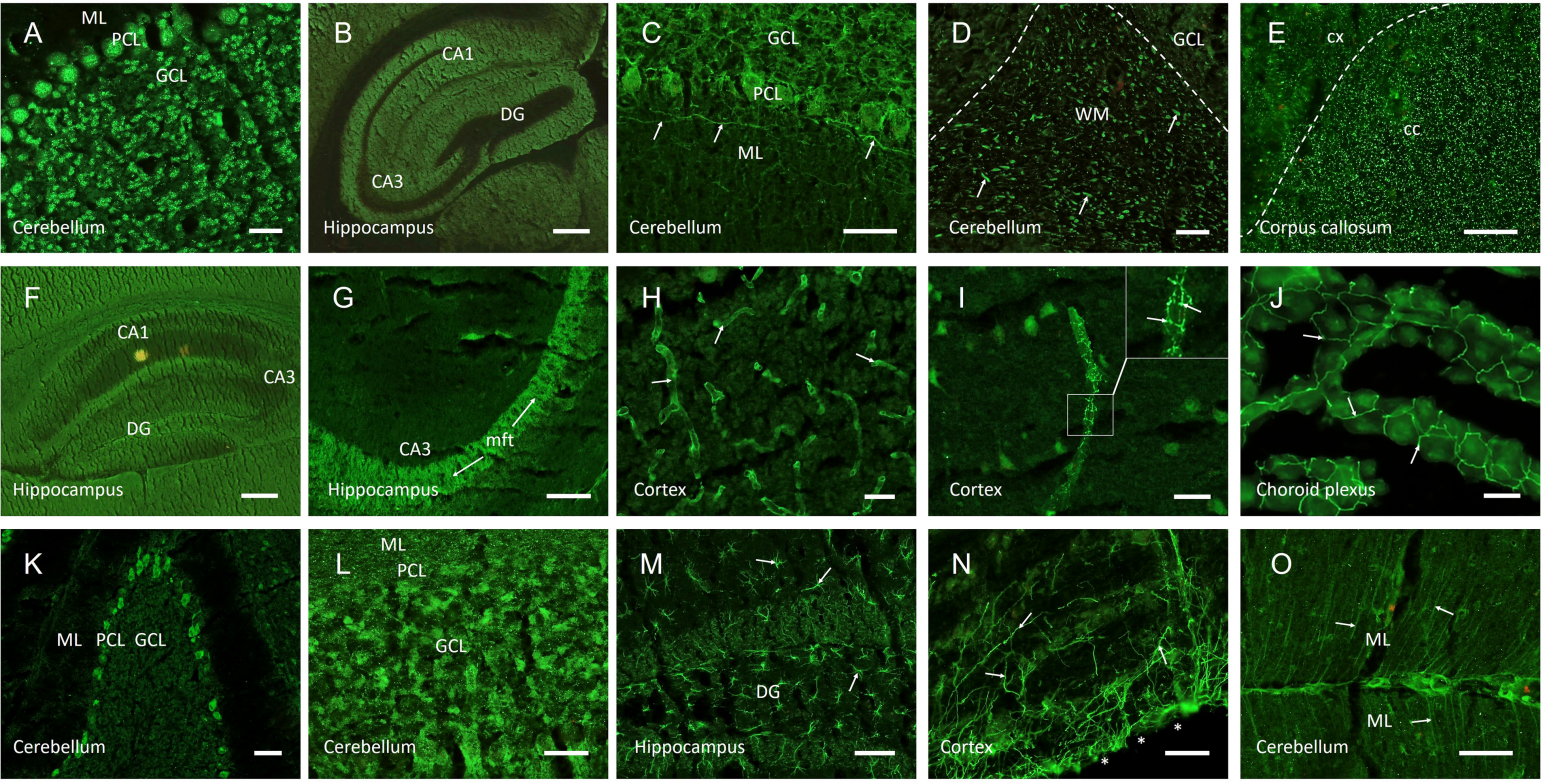
Representative images of autoantibody patterns using indirect immunofluorescence on unfixed mouse brain sections. CSF of patients with various neurologic and psychiatric symptoms demonstrated strong IgG autoreactivity to circumscribed anatomical structures. Samples with binding to anti-nuclear structures **(A)** were excluded from analysis, as were characteristic CSF probes with autoantibodies that were identified in parallel commercial CBAs, such as against NMDA receptors **(B)**. Anti-myelin autoantibodies ranged from neurofilament-like staining in the cerebellum (**C**, arrows) to cerebellar myelinated fibers (**D**, arrows) to punctated white matter stainings in the corpus callosum **(E)**. Neuropil staining was seen in larger anatomical areas such as the hippocampus **(F)**, but also in distinct sub-regions such as the hippocampal mossy fiber tract (**G**, arrows). Anti-vessel antibodies bound to cerebral blood vessels of various calibers (**H**, arrows), but also selectively to nervi vasorum (**I**, arrows in higher magnification image). Tight junction antibodies were observed in the choroid plexus (**J**, arrows). Anti-cellular patterns included staining of Purkinje cells **(K)** or granule cells in hippocampus or cerebellum **(L)**. Anti-astroglial antibodies reacted with astrocytes throughout the brain (**M**, arrows) or bound selectively to glia limitans (**N**, arrows, surface of the brain marked with asterisks) or Bergmann glia cells (**O**, arrows). Scale bars: **(A, C-D, H-L)** = 50 µm, **(E, M-O)** = 100 µm, **(B, F-G)** = 200 µm. ML, molecular layer; PCL, Purkinje cell layer; GCL, granule cell layer; DG, dentate gyrus; CA1/3, cornu ammonis 1/3; WM, white matter; cc, corpus callosum; cx, cortex; mft, mossy fiber tract.

The remaining 55 patients (20%) harbored CSF autoantibodies with novel immunofluorescence patterns and were followed up in detail. Most patients had suspected encephalitides (previously classified as ‘seronegative’), paraneoplastic or inflammatory neurological diseases, but also neurodegenerative diseases and syndromes of unknown etiology (**Figure 1B**). CSF autoantibodies targeted various brain regions and anatomical structures and were categorized into six main groups (**Table 1**, **Figure 2**):

1. myelin (n=13), typically in the cerebellar white matter (**Figure 2D**) or corpus callosum;
2. neuropil (n=14), often in basal ganglia, hippocampus (**Figures 2F-G**), cerebellum, or olfactory bulb;
3. blood vessels (n=8), including staining of endothelium (**Figure 2H**) or nervi vasorum (**Figure 2I**);
4. tight junctions (n=5), observed in the choroid plexus (**Figure 2J**);
5. cellular pattern (n=8), including soma and proximal dendrites of Purkinje cells (**Figure 2K**) or granule cells in cerebellum (**Figure 2L**) or hippocampus, and
6. astroglial proteins (n=7), including white matter astrocytes (**Figure 2M**), glia limitans (**Figure 2N**), or Bergmann glia cells (**Figure 2O**).

**Table 1:** Comparison of clinical, laboratory, treatment, and outcome profiles among the antibody groups.

	Total	Anti- myelin	Anti- neuropil	Anti- vessel	Anti-tight junction	Anti- cellular	Anti- astroglia
N°	55	13	14	8	5	8	7
Diagnosis							
AE/AP	31 (56)	6 (46)	7 (50)	5 (62)	1 (20)	6 (75)	6 (86)
PNSa)	5 (15)	1 (8)	2 (14)	–	2 (40)	–	–
Inflammatory	8 (7)	2 (15)	3 (21)	–	1 (20)	2 (25)	–
Neurodegenerative	4 (4)	1 (8)	1 (7)	2 (25)	–	–	–
Infectious	2 (9)	1 (8)	1 (7)	–	–	–	–
Unclassified	5 (9)	2 (15)	–	1 (12)	1 (20)	–	1 (14)
Demographics							
Age of onset	50 [38-58]	49 [32-51]	55 [42-49]	40 [38-52]	59 [53-65]	42 [20-61]	41 [36-63]
Female sex	29 (53)	3 (23)	8 (57)	5 (63)	4 (80)	6 (75)	3 (43)
Clinical profile							
Onset to final diagnosis (m)	4 [1-11]	2 [1-24]	6 [1-9]	1 [0-2]	4 [3-10]	10 [4-23]	1 [0-4]
Onset to antibody screening (m)	3 [0-10]	2 [0-23]	5 [1-8]	1 [0-1]	4 [3-9]	10 [4-32]	1 [0-4]
Initial mRS	4 [3-4]	4 [3-4]	4 [3-4]	3 [3-4]	3 [3-5]	4 [3-4]	4 [3-5]
Comorbidities							
Autoimmune	4 (7)	–	2 (14)	–	1 (20)	1 (13)	–
Neoplasia (present/past)	10 (18)	2 (15)	2 (14)	–	2 (40)	2 (25)	2 (29)
Infectious	12 (22)	3 (23)	3 (21)	1 (13)	–	4 (50)	1 (14)
Neurologic	20 (36)	3 (23)	3 (21)	4 (50)	1 (20)	3 (38)	6 (86)
Psychiatric	15 (27)	3 (23)	**-**	3 (38)	1 (20)	2 (25)	3 (43)
**Brain MRI**	55	13	14	8	5	8	7
Normal	18 (33)	1 (8)	4 (29)	4 (50)	3 (60)	4 (50)	2 (29)
Pathological	37 (67)	12 (92)	10 (71)	4 (50)	2 (40)	4 (50)	5 (71)
Atrophy	6 (11)	–	2 (14)	–	1 (20)	2 (25)	1 (14)
Lesions	30 (55)	12 (92)	7 (50)	4 (50)	1 (20)	2 (25)	4 (57)
Supratentorial	29 (53)	11 (85)	7 (50)	4 (50)	1 (20)	2 (25)	4 (57)
Infratentorial	9 (16)	4 (31)	3 (21)	1 (13)	–	–	1 (14)
**Spinal MRI**	9	2	4	–	–	–	3
sMRI, normal	5 (56)	2 (100)	1 (25)	–	–	–	2 (67)
sMRI, lesions	4 (44)	–	3 (75)	–	–	–	1 (33)
**EEG**	51	12	12	8	5	7	7
EEG normal	34 (62)	9 (75)	8 (67)	4 (50)	3 (60)	6 (86)	4 (57)
Pathological EEG	17 (33)	3 (25)	4 (33)	4 (50)	2 (40)	1 (14)	3 (43)
Epileptic signals	14 (27)	2 (17)	3 (25)	3 (38)	2 (40)	1 (14)	3 (43)
General slowing	3 (6)	1 (8)	1 (8)	1 (13)	–	–	–
CSF							
Cell count [<5/µl]^b^)	4 [2-18]	12 [2-15]	3 [1-12]	3 [1-18]	2 [2-3]	2 [2-6]	83 [14-128]
Glucose [<70 mg/dl]	64 [58-72]	62 [60-70]	62 [58-74]	69 [58-83]	67 [65- 69]	64 [58-71]	59 [50-64]
Lactate [<22mg/dl]	15 [14-18]	15 [14-20]	16 [15-18]	14 [14- 16]	15 [13- 15]	16 [14-17]	16 [14-22]
Protein [<450 mg/dl]	398 [307 -652]	518 [400 -923]	388 [308 -490]	322 [229 -778]	316 [316 -333]	378 [268 -514]	438 [298- 912]
CSF-restricted OCB (%)	21 (38)	7 (54)	7 (50)	2 (25)	1 (20)	1 (13)	3 (43)
**Neurofilament**	23	6	7	1	3	3	3
Neurofilament[<827	2272 [615-	2647 [591-	2912 [625-	332	2333 [644-	583 [51-	7699 [7699-
pg/ml]	4383]	5406]	4676]		2990]	1014]	8978]
Outcome profile									
Duration follow-up (m)	8 [4-12]	11 [3-12]	7 [6-11]	13 [2-36]	21 [21-21]	6 [4-11]	7 [6-8]
***With treatment***	46 (84)	11 (85)	12 (86)	6 (75)	4 (80)	6 (75)	7 (100)
*Cohort 1*	21 (46)	6 (55)	4 (33)	3 (50)	2 (50)	4 (67)	2 (29)
*Cohort 2*	25 (54)	5 (45)	8 (67)	3 (50)	2 (50)	2 (33)	5 (71)
Initial mRS	4 [3-4]	4 [3-4]	4 [3-4]	3 [3-4]	4 [3-5]	4 [3-5]	4 [3-5]
Last mRS	3 [1-3]	2 [2-4]	2 [1-3]	3 [2-3]	3 [2-3]	2 [1-3]	3 [2-3]
Favorable mRS	21 (46)	6 (55)	6 (50)	2 (33)	1 (25)	3 (50)	3 (43)
***Without treatment***	9	2	2	2	1	2	–
Initial mRS	3 [3-3]	2 [2-3]	2 [2-2]	4 [4-4]	3 [3-3]	3 [3-3]	–
Last mRS score	2 [2-3]	2 [1-2]	2 [2-3]	4 [4-4]	2 [2-2]	3 [2-3]	–
Favorable mRS	5 (56)	2 (100)	1 (50)	–	1 (50)	1 (50)	–
Treatment profile									
Onset to therapy (m)	5 [1-10]	2 [1-24]	6 [1-9]	1 [0-2]	4 [3-10]	6 [2-17]	1 [0-4]
Duration of therapy (m)	7 [3-29]	4 [3-12]	26 [8-37]	6 [5-30]	4 [4-5]	8 [3-26]	6 [3-9]
First-line therapy	46 (84)	11 (85)	12 (86)	6 (75)	4 (80)	6 (75)	7 (100)
Second-line therapy	22 (40)	5 (39)	7 (50)	3 (38)	–	3 (38)	4(57)
Third-line therapy	3 (6)	1 (8)	1 (7)	1 (13)	–	–	–
Tumor therapy^c)^	5 (9)	1 (8)	2 (14)	–	2 (40)	–	–

Data are presented as median [interquartile range] or frequency (%) as appropriate. ^a)^ Breast cancer (n=2), lymphoplasmacytic lymphoma (n=1) and Merkel cell carcinoma (n=1). ^b)^ Tumor therapy is only related to patients with paraneoplastic neurologic syndrome and does not consider all tumor treatments of patients with neoplastic diseases in the past. ^c)^ All patients (with normal and increased white blood count) showed a lymphocytic cell pattern in the CSF.

AE/AP, autoimmune encephalitis/ autoimmune psychosis; CSF, cerebrospinal fluid; EEG, electroencephalography; m, months; OCB= oligoclonal bands; PNS, paraneoplastic neurologic syndrome; y, years

Serum was available from 43 of the 55 patients (78%), of whom 26 (60%) had the identical pattern in both CSF and serum, while 17 (40%) were exclusively positive in CSF.

### Clinical findings

Initial clinical presentations of the CNS-Ab-positive 55 patients included epileptic seizures (n=13), cognitive impairment (n=12), encephalopathy (n=9), cerebellar symptoms (n=8), neuromuscular symptoms (n=6), psychiatric manifestations (n=6), autonomic symptoms (n=4), and visual symptoms (n=2). Autoimmune encephalitis was the most common diagnosis and occurred across all Ab groups. The median age at onset was 50 (IQR 38-58) and 53% of patients were female (29/55). The median time from symptom onset to Ab screening was 3 months (IQR 0-10), one third of patients was referred for a second opinion. Details of clinical presentation, comorbidities including cancer, MRI (abnormalities in 67%), EEG (abnormalities in 33%) and CSF findings (e.g., oligoclonal bands in 38%), (immuno)therapies and outcome (assessed by mRS) are presented in **Table 1**. Neurodegeneration markers (t-Tau, p-Tau, Aβ42, Aβ42/40 ratio) were assessed in 19 of 55 patients (35%) and pathological in four cases, all diagnosed with neurodegenerative diseases. NfL levels in CSF (measured in 23 of 55 patients (42%)) were markedly increased (median 2272 pg/mL [IQR 615–4383]), particularly in patients with anti-myelin, anti-neuropil, anti–tight junction, and anti-astroglial IgG reactivity.

We did not identify clinical parameters or biomarkers that were – at these sample sizes – significantly associated with the presence of a specific Ab pattern (**Table 1**, **Supplementary Figure 1**). *Myelin-specific Abs* were frequently associated with memory impairment (77%) and gait disorders (62%), supratentorial MRI lesions (84%), mild pleocytosis (median 12/µl, IQR 2-18) and CSF-restricted OCBs (54%). Patients with *neuropil Abs* more often had gait disability (71%), language problems (64%) and motor weakness (57%), CSF-restricted OCBs were common (50%). Immunotherapies were administered much longer than in patients with other Abs (median 26 months, IQR 8–37, compared to 6 months, IQR 3–12). Abs reacting against *blood vessels* of different calibers (including small, medium, and large vessels) were predominantly seen in patients with cognitive impairment (88%) and psychiatric symptoms (75%). *Abs against tight junctions* most often presented with memory impairment (80%). In patients with *anti-cellular Abs*, psychiatric symptoms were the leading symptom (88%), followed by memory impairment (63%). Half of these patients received the diagnosis of possible psychiatric AE and the time from disease onset to Ab screening was much longer (median of 10 months, IQR 4-32) compared to the other Ab groups (2 months, IQR 0-8). All patients with *astroglial Abs* exhibited memory impairment (100%) and many psychiatric symptoms (86%), most patients had elevated CSF cell counts (71%, median of 83 cells/µl, IQR 3-150). After detection of an astroglial tissue pattern, samples were tested for the presence of GFAP Abs (which were not included in our initial panel of established autoantibodies), turning out positive in 5/7 cases and clinically matching the known spectrum of GFAP astrocytopathy.

**Figure 3:**
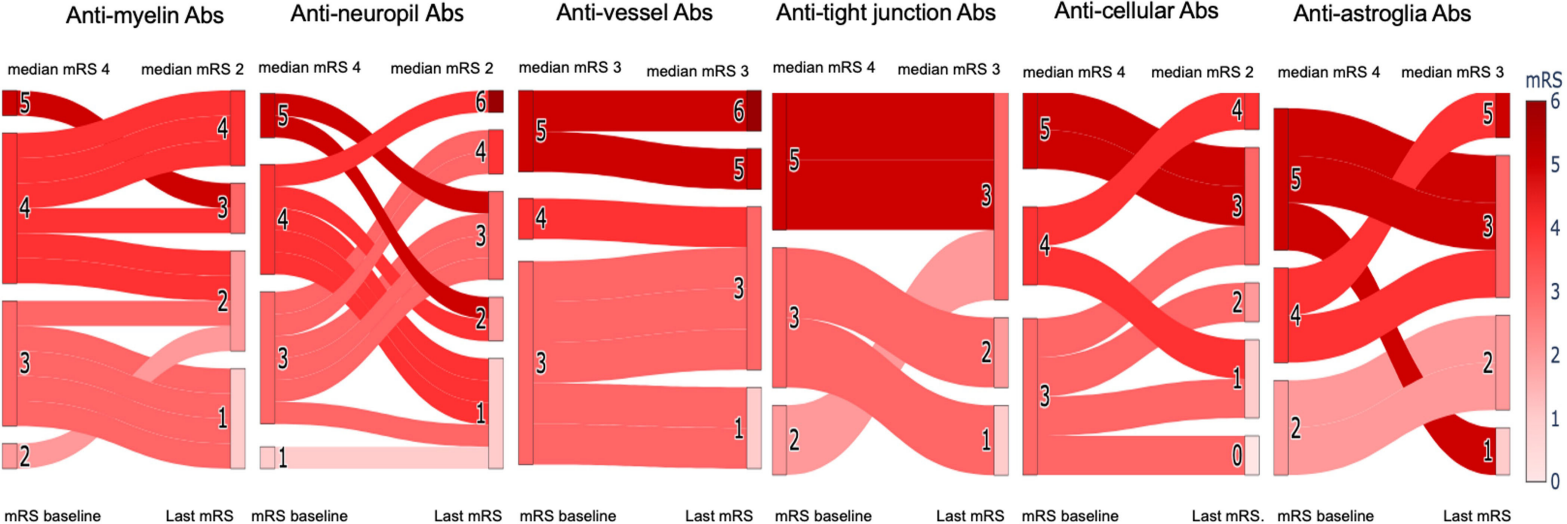
Clinical outcomes stratified by antibody profiles. Changes in the modified ranking scale (mRS) score for each autoantibody subtype from baseline (=start of immunotherapy) to last follow-up (median 8 months). Kruskal-Wallis test was performed to compare the groups, revealing no significant differences.

Given that the differences between Ab groups require validation in larger cohorts, we nonetheless observed variable responses to immunotherapy (**Figure 3**). Patients with Abs against myelin, neuropil, and cellular structures responded well to immunotherapy, with a median mRS reduction from 4 to 2 points. Moderate improvement (median mRS reduction by 1 point) was seen in patients with anti-astroglial and anti-tight junction Abs, while no improvement was observed in patients with anti-vessel Abs.

### Novel CNS-Abs support identification of immunotherapy-responsive patients

We next asked whether a positive Ab finding resulted in changes in clinical management of patients, in particular in the administration of immunotherapies. Of the 55 Ab-positive patients, 9 did not receive immunotherapy. These patients were finally diagnosed with neurosyphilis (n=1), monophasic demyelinating disease of unknown etiology (n=1), frontotemporal dementia-amyotrophic lateral sclerosis FTD-ALS) (n=1), developmental disorder and paranoid schizophrenia (n=1), vascular dementia (n=1), mild cognitive impairment (MCI) after herpes encephalitis (n=1), Creutzfeldt-Jakob disease (n=1), schizoaffective disorder (n=1), and a combination of bradykinetic syndrome, tremor, MCI, and chronic fatigue of unknown etiology (n=1).

**Figure 4:**
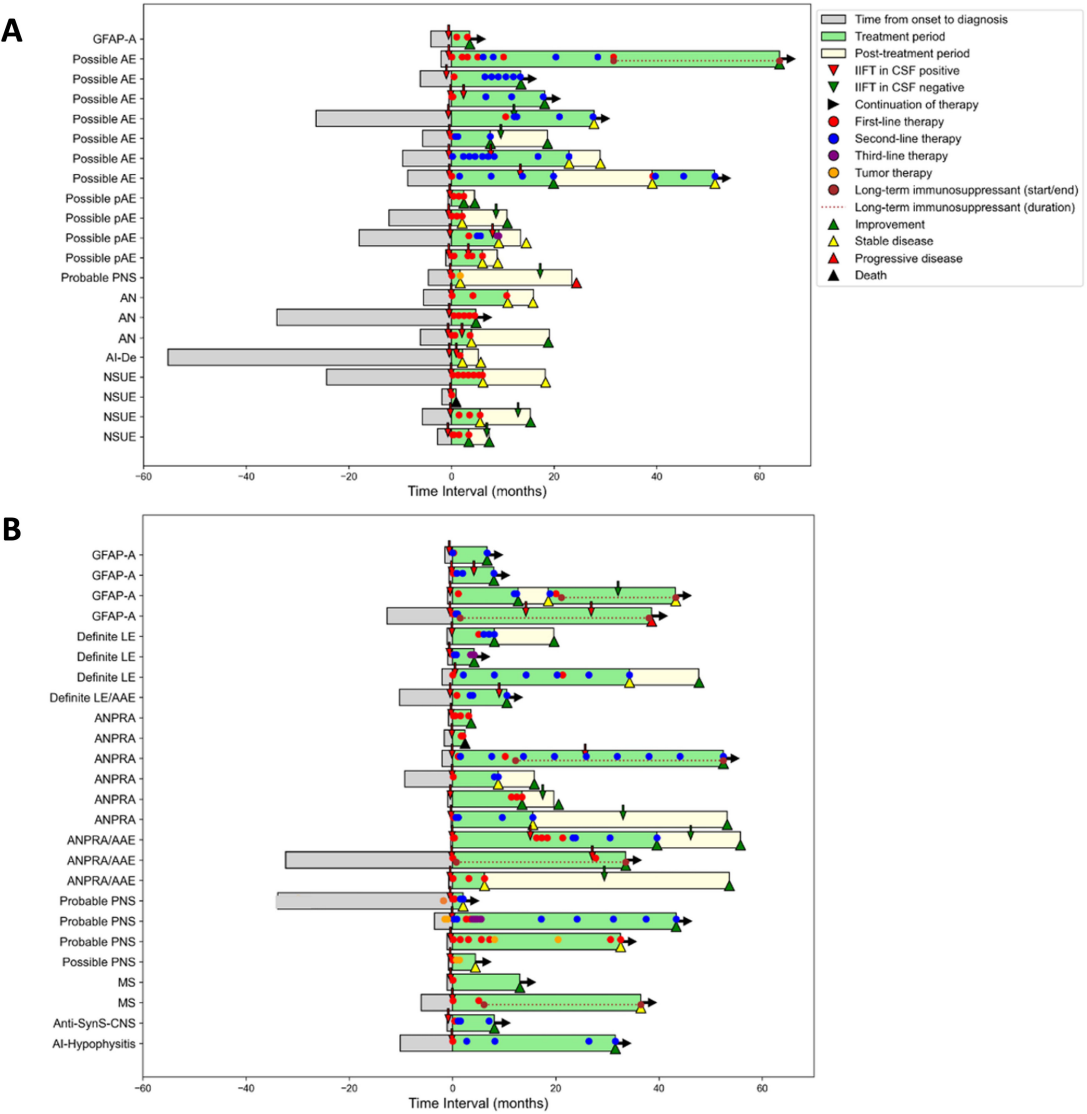
Clinical and treatment history in CSF autoantibody-positive patients Comparison of the medical history of patients in whom the decision for immunotherapy based exclusively on positive autoantibodies in TBA (**A,** *cohort 1*) versus those who would have been treated also without TBA positivity due to inflammatory signs in MRI or CSF (**B,** *cohort 2*). Each bar represents one patient and shows time from symptom onset to diagnosis, treatment initiation (time point 0), treatment duration and post-treatment period, type of treatment, and outcome (improvement = reduction of ≥1 mRS point, stable disease = no mRS change, deterioration = increase of ≥1 mRS point on the mRS). AAE, autoimmune associated epilepsy; AI-Hypophysitis, autoimmune hypophysitis; AN, suspected autoimmune neuropathy; ANPRA, autoantibody-negative but probable autoimmune encephalitis; Anti-SynS-CNS, Anti-synthetase syndrome with central nervous system involvement; GFAP-A, GFAP astrocytopathy; IIFT, indirect immunofluorescence testing; LE, limbic encephalitis; MS, multiple sclerosis; AI-De, suspected autoimmune dementia; NSUE, neurological syndrome of unknown etiology; PNS, paraneoplastic neurological syndrome.

The remaining 46 patients received immunotherapy at the discretion of the treating physicians (**Figure 4**). In 21 (46%) of these, immunotherapy was initiated only because of the positive finding of novel CNS Abs in the TBA (*Cohort 1*, **Figure 4A**). Characteristic constellations included patients with the working diagnosis of possible AE (n=7) or possible autoimmune psychosis (n=4), but no abnormalities in routine MRI and CSF parameters. Likewise, patients with suspected autoimmune neuropathies (n=3) (previously classified ‘suspected motor neuron disease’) and neurological syndromes of unknown etiology such as unclassified ataxia, MCI, muscle weakness, or autonomic dysfunction (n=4), were only treated after recognition of CNS Abs.

In 25 patients (54%), immunotherapy would have been initiated also without knowledge of the CNS Abs due to other evidence of neuroinflammation (*Cohort 2*, **Figure 4B**). Diagnoses included LE (n=4), ‘seronegative’ probable autoimmune encephalitis (n=10) or possible/probable paraneoplastic neurological syndrome (n=2). In all these patients of cohort 2, additional findings already supported an autoimmune etiology, e.g. brain MRI compatible with demyelination or inflammation, systemic autoimmune disease with suspected CNS affection, or inflammatory CSF.

In patients of cohort 1, the duration between symptom onset and initiation of Ab diagnostics was significantly delayed compared to cohort 2, which resulted in a similar delay in the final diagnosis (median 5 months, IQR 2-12, compared to 1 month, IQR 0-6; p=0.0125) (**Table 2**).

**Table 2:** Comparison of treatment timelines of cohort 1 and cohort 2.

	Total n=46	Cohort 1 n=21	Cohort 2 n=25	P-value
Onset to diagnosis (m)	2 [1-9]	5 [2-12]	1 (0-6]	0.01*
Onset to Ab screening (m)	2 [0-8]	5 [2-11]	0 [0-5]	0.01*
Onset to therapy	5 [1-10]	6 [2-12]	2 [0-10]	0.07
Duration of treatment (m)	7 [3-29]	5 [2-12]	9 [4-33]	0.06
Treatment profiles ^a)^				
First-line therapy	46 (100)	21 (100)	25 (100)	–
Steroids	38 (83)	15 (71)	23 (92)	0.12
IVIG	17 (37)	9 (43)	8 (32)	0.45
PE/IA	25 (54)	11 (52)	14 (56)	0.81
Second line therapy	22 (48)	6 (29)	16 (64)	0.02*
Rituximab	16 (35)	4 (19)	12 (48)	0.04
Cyclophosphamide	3 (7)	2 (10)	1 (4)	0.59
Long Term IST ^b)^	7 (15)	1 (5)	6 (24)	0.11
Third-line therapy	3 (7)	1 (5)	2 (8)	1.00
Daratumumab	2 (4)	–	2 (8)	0.49
Bortezomib	1 (2)	1 (5)	–	–
Tumor therapy	5 (100)	1 (5)	4 (16)	0.36

Data are presented as n (%) for categorical variables and as median (interquartile range [IQR]) for continuous variables. Statistical comparisons were performed using the Mann–Whitney U test or an unpaired t-test, as appropriate. *P < 0.05. ^a)^ Patients may appear in multiple therapy groups concurrently. b) Long term IST included azathioprine, mycophenolate mofetil, methotrexate. AB, autoantibody; IA, immunoadsorption; IST, long-term immunosuppressive treatment; IVIG, intravenous immunoglobuline; m, months; PE, plasma exchange.

Treatment was initiated later, administered for a shorter period of time, and escalation to second-line treatment was less likely in cohort 1. Despite these differences, the vast majority of patients in both cohorts improved (cohort 1: 52% vs. cohort 2: 76%; OR 0.35, 95% CI 0.10–1.22, p = 0.09) or stabilized (cohort 1: 38% vs. cohort 2: 12%; OR 4.5, 95% CI 1.01–20.10, p = 0.04) at follow-up (**Table 3**), indicating that consideration of the novel Abs helped to identify (and treat) immunotherapy-responsive patients in clinical routine—even in the absence of classical inflammatory markers.

**Table 3:** Comparison of outcome profiles between cohort 1 and cohort 2.

	Total n=46	Cohort 1 n=21	Cohort 2 n=25	OR (CI 95%), p-value
Outcome profiles				
Improvement	30 (65)	11 (52)	19 (76)	0.35 (0.10-1.22), 0.09
Stable disease	11 (24)	8 (38)	3 (12)	4.5 (1.01-20.10), 0.04
Deterioration/Death	5 (11)	2 (10)	3 (12)	0.77 (0.12-5.12), 1.00
Favorable outcome	21 (46)	8 (38)	13 (52)	0.57 (0.18-1.85), 0.35

Outcomes refer to the last available follow-up (median 8 months). Odds ratios (OR) with 95% confidence intervals (CI) and p-values are shown for comparisons. Statistical analyses were performed using the Mann–Whitney U test or an unpaired t-test, as appropriate.

In 22 of the 46 treated cases (48%), Ab testing in CSF using TBA was repeated. Of 14 patients re-tested during the treatment period, 12 had persistent Ab findings while two turned negative. Of 10 patients re-tested in the follow-up period post-treatment, all of them were Ab-negative, accompanied by clinical improvement or stabilization in nine cases. Although not formally assessed, loss of CNS Abs may therefore help to monitor clinical responses and cessation of immunotherapy.

## Discussion

In this study, we identified novel CNS Abs in the CSF of 20% of patients with suspected autoimmune neuropsychiatric conditions who tested negative for established Abs in routine diagnostic panels. Using unfixed murine TBAs, we defined six distinct Ab groups: anti-myelin, anti-neuropil, anti-vessel, anti-tight junction, anti-cellular, and anti-astroglial Abs. Clinical symptoms were heterogeneous, with memory impairment (73%) and psychiatric symptoms (64%) being the most frequent manifestations. Immunotherapy was administered to most patients, however, in 46% decision for treatment initiation was based on the presence of the novel CNS Abs, indicating that TBA results can guide the identification of immunotherapy-responsive patients. In support of this, follow-up Ab testing demonstrated disappearance of Abs parallel to clinical improvement.

The results of this study highlight the potential clinical relevance of extensive Ab testing including TBAs in neuropsychiatric disorders and support related studies (13). Remarkably, novel CNS Abs in TBAs were detected twice as often as those with known Abs, demonstrating their potential value not only as a screening tool but also in identifying patients with yet uncharacterized or emerging Abs – thus helping to close a current diagnostic gap (3, 14, 15). In several cases, initiation of immunotherapy based on TBA findings was further supported by markedly elevated CSF NfL levels, indicating ongoing axonal damage possibly linked to antibody-mediated processes. This aligns with the established role of NfL as a broadly applicable biomarker of neuroaxonal injury, whose clinical relevance has been demonstrated across a broad range of neurological diseases – including inflammatory, degenerative and vascular etiologies (28). However, these Ab findings should always be interpreted in clinical context, and alternative diagnoses should be thoroughly considered and excluded.

Novel CNS autoantibodies may in the future facilitate earlier initiation of immunotherapy, allow monitoring of disease activity, and improve prognosis. Towards this goal, it is of utmost importance to identify the underlying targets and add the antigens to diagnostic panels (29), which can then become widely available. Likewise, cloning, and recombinant production of patient-derived monoclonal human antibodies will help to elucidate the underlying disease mechanisms *in vitro* and in animal models. Results of such experiments will be particularly helpful to determine which of the here described TBA patterns correspond to pathogenic autoantibodies, and which are non-pathogenic binders that would not implicate the need for immunotherapy.

Memory impairment and psychiatric symptoms emerged as predominant features across Ab subgroups in this study, consistent with the growing recognition that autoimmune CNS disorders can present as cognitive and behavioral syndromes (3, 30). Depending on the underlying antigens, Abs have the potential to contribute to pathology across several diseases, where they not necessarily cause the entire disease, but shape its symptomatology and underlying pathogenesis, comparable to other modulators, such as genetics, lifestyle, metabolic or environmental risk factors (3). Along these lines, we previously detected comparable and overlapping brain immunofluorescence patterns in other clinical conditions, ranging from AE (22) and COVID-19 disease with neurological symptoms (16) to various dementia types (31) and seizures of unknown etiology (32). TBA findings can support clinical decision-making also in psychiatric conditions, such as depression (33–35), schizophreniform and affective disorders (17) and obsessive-compulsive disorders (36). It is important to note that other studies reported less frequent findings of Abs in psychiatric diseases such as first-episode psychosis, potentially related to different biosamples (serum rather than CSF), use of cell-based assays or fixed tissues (37–39).

In our cohort, 40% of Abs were detected exclusively in CSF, strengthening the low-threshold assessment of CSF in unexplained neuropsychiatric diseases. Restriction of Abs to the CNS compartment is also know from other autoimmune diseases, such as in 14% of patients with NMDAR encephalitis (40). Early work already demonstrated that Ab detection may have higher sensitivity in CSF, associate with clinical phenotypes and has consistent results across various studies and laboratories (41). We therefore focused on the clinical, diagnostic, and therapeutic relevance of novel Ab findings in CSF.

Despite increasing awareness of the clinical relevance of Ab testing in neuropsychiatric diseases and integration into clinical practice guidelines (19, 42), identifying patients in time for extended screening remains challenging in routine care. In our study, mean initiation of immunotherapy started several months after symptom onset. Factors contributing to this delay may relate to atypical clinical presentations and normal findings in CSF or MRI. Neurologists had to learn over the last years, that even well-established AE forms can have normal CSF and MRI findings, such as in the majority of patients with IgLON5 disease or LGI1 encephalitis (43). In the same way, some of the here described novel CNS Abs may become “well-established” in the future. In fact, the here identified astroglial Abs turned out to represent GFAP Abs in most cases and were associated with the clinical picture of GFAP encephalopathy. They were not detected with CBAs as this is still not part of the routine screening panel in our center (and in most hospitals worldwide). Such findings challenge the common term “seronegative AE”, as the term is frequently used when only a limited number of autoantibodies have been excluded, while extensive screening including TBAs on unfixed brain sections are not always considered. We argue that the presence of certain TBA-based Abs, in particular if found in CSF, should prompt consideration of immunotherapy already today, given also that many first-line and second-line treatments are well tolerated. At the same time, prospective, multicenter studies involving larger cohorts are essential to validate the prevalence of these Abs across various neuropsychiatric conditions and to establish their utility as diagnostic and prognostic biomarkers.

The study has several limitations. The retrospective design and single-center setting may introduce selection bias, as patients with severe or atypical presentations were more likely to undergo extended Ab testing. The (obvious) lack of CSF samples from healthy controls limits our ability to distinguish pathogenic Abs from incidental findings. Likewise, there was no Ab-negative control group as matching for similar symptoms or diseases was not possible. Additionally, the small sample sizes within individual Ab groups constrain statistical power and the generalizability of our findings. Therefore, estimating the prevalence of novel CSF Abs across various neurological disorders is not feasible. Also, technical aspects of the TBA have to be considered. Handling of unfixed mouse brain requires rigorous standard operating procedures, but is still prone to artifacts, which we try to control by repetition of stainings. A given immunofluorescence patterns may further result from Abs to very different antigens, which cannot be separated in TBAs.

Taken together, this study indicates diagnostic and therapeutic relevance of novel CNS Abs in patients with neuropsychiatric disorders, particularly in those classified as ‘seronegative’ AE or psychiatric autoimmune presentations. Parallel assessment using TBAs can help to identify immunotherapy-responsive cases and should therefore be applied at low threshold in clinical routine. Future research should validate the clinical relevance of these findings, identify the underlying targets, and elucidate their pathogenic roles. Incorporating these approaches into routine diagnostic workflows may improve the recognition and treatment of autoimmune neuropsychiatric disorders, ultimately enhancing patient outcome.

## Data Availability

The raw data supporting the conclusions of this article will be made available by the authors, without undue reservation.
