# Supplementary material for: Novel cerebrospinal fluid anti-central nervous system IgG antibodies can identify immunotherapy-responsive neuropsychiatric disorders
Source: Front Immunol. 2025 Jul 7;16:1612844. doi: 10.3389/fimmu.2025.1612844 (PMC12277280; doi:10.3389/fimmu.2025.1612844)
Supplement: Supplementary file 1 [file Table1.docx]

**Supplementary Information**

Supplementary material 1:


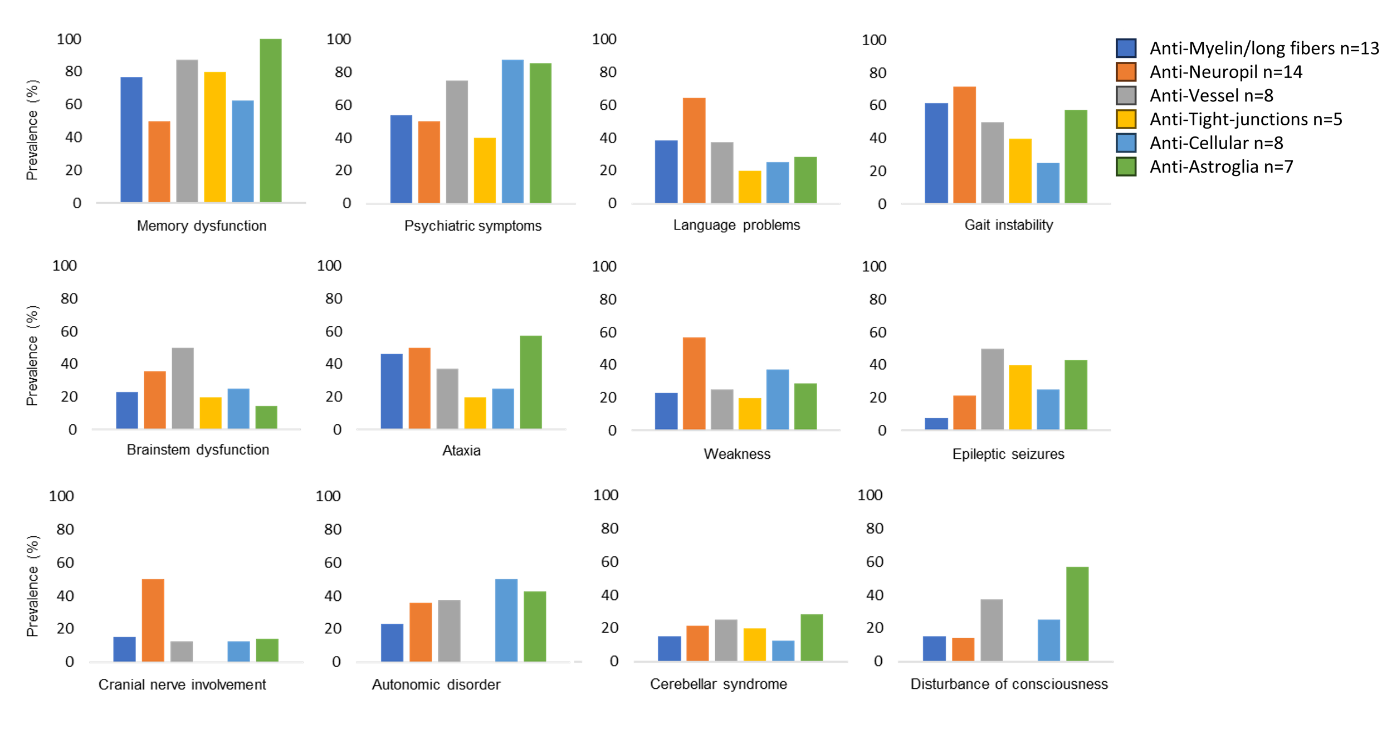


**Figure S1. Prevalence of neurological and psychiatric symptoms in patients with novel anti-CNS antibodies categorized by immunofluorescence-defined antibody groups.**

Antibody groups were defined according to distinct IgG binding patterns on unfixed murine brain tissue, including anti-myelin (n=13), anti-neuropil (n=14), anti-vessel (n=8), anti-tight junction (n=5), anti-cellular (n=8) and anti-astroglial (n=7) reactivities. Symptom prevalence was compared between groups using Fischer`s exact test. No statistically significant group differences were observed (p> 0.05).
